# Mechanisms Underlying the Cognitive and Behavioural Effects of Maternal Obesity

**DOI:** 10.3390/nu13010240

**Published:** 2021-01-15

**Authors:** Kyoko Hasebe, Michael D. Kendig, Margaret J. Morris

**Affiliations:** Department of Pharmacology, School of Medical Sciences, UNSW Sydney, Sydney, NSW 2052, Australia; k.hasebe@unsw.edu.au (K.H.); m.kendig@unsw.edu.au (M.D.K.)

**Keywords:** maternal obesity, cognition, behaviour, neuroinflammation, metabolism, gut microbiome

## Abstract

The widespread consumption of ‘western’-style diets along with sedentary lifestyles has led to a global epidemic of obesity. Epidemiological, clinical and preclinical evidence suggests that maternal obesity, overnutrition and unhealthy dietary patterns programs have lasting adverse effects on the physical and mental health of offspring. We review currently available preclinical and clinical evidence and summarise possible underlying neurobiological mechanisms by which maternal overnutrition may perturb offspring cognitive function, affective state and psychosocial behaviour, with a focus on (1) neuroinflammation; (2) disrupted neuronal circuities and connectivity; and (3) dysregulated brain hormones. We briefly summarise research implicating the gut microbiota in maternal obesity-induced changes to offspring behaviour. In animal models, maternal obesogenic diet consumption disrupts CNS homeostasis in offspring, which is critical for healthy neurodevelopment, by altering hypothalamic and hippocampal development and recruitment of glial cells, which subsequently dysregulates dopaminergic and serotonergic systems. The adverse effects of maternal obesogenic diets are also conferred through changes to hormones including leptin, insulin and oxytocin which interact with these brain regions and neuronal circuits. Furthermore, accumulating evidence suggests that the gut microbiome may directly and indirectly contribute to these maternal diet effects in both human and animal studies. As the specific pathways shaping abnormal behaviour in offspring in the context of maternal obesogenic diet exposure remain unknown, further investigations are needed to address this knowledge gap. Use of animal models permits investigation of changes in neuroinflammation, neurotransmitter activity and hormones across global brain network and sex differences, which could be directly and indirectly modulated by the gut microbiome.

## 1. Background

The worldwide prevalence of obesity has tripled since 1975, accompanied by a sharp increase in obesity among women of reproductive age [1]. Consumption of so-called ‘western’ diets high in saturated fat and sugar, along with more sedentary lifestyles have contributed to the global epidemic of obesity. Environments geared to maximise consumption of palatable food encourage excess energy intake, which confers risk of metabolic and cognitive impairment in adults that can be transferred to the next generation via a suite of molecular and epigenetic mechanisms [2]. Maternal obesity imposes increased risk to maternal health which extends to the health and mental wellbeing of their offspring. Increasing evidence from epigenetic, clinical and preclinical studies shows that maternal obesity from pre-pregnancy to lactation affects offspring neurodevelopment, and increases the risk of behavioural and emotional problems including delayed or impaired psychosocial behaviour, hyper-reactivity, autism spectrum disorder, anxiety and cognitive impairment [3,4,5,6,7].

Observations regarding neurodevelopmental impacts of maternal obesity have emerged more recently, building on extensive evidence linking higher maternal body mass index (BMI) to adverse offspring metabolic and cardiovascular outcomes. This literature has been reviewed elsewhere and is not examined here [5,8,9]. Rather, this review provides an overview of the mechanisms underlying the effects of maternal obesity on offspring emotional and cognitive function, with a focus on the central nervous system (CNS) changes observed in offspring in rodent models of maternal obesity. We evaluate the consistency of results from preclinical models with the available evidence from human data and suggest directions for future research. Relevant literature was located on PubMed between July–August 2020 using the following terms: (pregnancy, prenatal, antenatal or prenatal) AND (Nutrition, Nutrient and Diet) AND (Neurodevelopment OR Brain OR Cognition OR Mental health). Literature from the last decade (2010–2020) was screened by abstract; studies that did not report brain or behavioural outcomes in offspring were excluded. Only studies in which offspring were weaned onto control diets are included in summary Table 1; Table 2. However, other relevant literature is discussed in relation to the underlying mechanisms by which maternal overnutrition impacts offspring.

## 2. Cognitive & Behavioural Effects of Maternal Obesity/Maternal Overnutrition on Offspring

Human and preclinical studies of the impacts of maternal obesity and overnutrition on offspring behaviour can be categorised into three areas: cognition, affective states and psychosocial behaviour. Although human studies have shown associations between these domains and maternal body weight and diet (see summary of clinical studies in Table 1), identifying the underlying neurobiological mechanism/s is challenging in human studies. Hence, preclinical studies are essential for providing mechanistic insight. Table 2 summarises relevant preclinical models of maternal overnutrition and obesity from the past decade, in which offspring were weaned onto a control diet. This is an important manipulation that is not feasible in human studies and which allows for the influence of maternal obesity to be more clearly isolated. The behavioural domains that have been most commonly assessed in rodent studies are cognition (memory and learning), affective state (anxiety- and depressive-like behaviours) and psychosocial behaviour (social recognition and interaction). 

### 2.1. Cognitive Function

#### 2.1.1. Human Studies

While maternal obesity has long been known to pose increased cardiometabolic risk for offspring, reviews and meta-analyses over the past decade have focused on the impacts of maternal obesity on offspring behaviour and cognition [5,50]. For instance, in a study of 1361 mother-child pairs (Project Viva cohort), Monthe-Dreze et al. [20] found that higher pre-pregnancy BMI (>30) was associated with poorer visual motor abilities at three years of age, with this relationship partly mediated by maternal inflammation (dietary inflammatory index and n−6:n−3 PUFA ratio). Another study of 2084 mother-child pairs in the Child Health Development Studies reported that maternal overweight (pre-pregnancy BMI 25–29.9) and obesity (pre-pregnancy BMI ≥ 30) were associated with lower verbal recognition scores at nine years of age [25]. While the degree of maternal obesity has been commonly assessed using pre-pregnancy BMI, it is important to note that maternal dietary patterns make independent contributions to child cognitive function. Higher maternal sugar sweetened beverage consumption was associated with lower cognitive functioning scores in early childhood, independent of pre-pregnancy BMI, in 1234 mother-child pairs in the Project Viva cohort [10]. While these studies indicate that maternal obesity and overnutrition negatively impact child cognitive development, environmental factors such as lifestyle, education and maternal mental state could also partly explain these associations. Hence, use of animal models to study such relationships under controlled environments, and where neuronal circuitries can be assessed, is essential to identify the underlying neurobiological mechanisms by which cognitive functioning is disrupted in offspring. 

#### 2.1.2. Preclinical Studies

We identified nine studies investigating the effects of maternal overnutrition (through high-fat, cafeteria-style and sucrose/fructose diet feeding) on offspring cognition. One of the challenges in interpreting behavioural data regarding cognition is the heterogeneity in tests and test parameters employed across studies, as noted in a recent meta-analysis and systematic review [51] which could limit translational relevance. As shown in Table 2, the neurobiological mechanisms most commonly invoked to explain these underlying behavioural changes include increased inflammatory state, altered neuronal plasticity and changes in brain metabolism. 

Increased proinflammatory signals including IL1β and TNFα were found in the whole brain of male and female mouse offspring of obese mothers at P0, with mixed effects seen at P7 and P21 [33]. Elevated lipid peroxidation markers including TBARS (thiobarbituricacid reactive substance), NADPH (nicotinamide adenine dinucleotide phosphate hydrogen) and SOD (superoxide dismutase) appeared as signs of oxidative stress in the hippocampus of rat offspring exposed to high sugar diets prenatally [46]. Neuronal changes are also reported; for instance, abnormal morphological changes in pyramidal neurons in the basolateral amygdala and CA1 region of the hippocampus in rat offspring of high fat diet (HFD)-fed dams [34]. Disrupted neuronal growth, decreased levels of BNDF and epigenetic changes in the hippocampus have been observed in rat and mouse offspring of mothers fed HFD [40,48]. Unsurprisingly, the altered neuronal phenotypes negatively affect neurotransmitter signalling, with altered function of the dopaminergic system in the striatum and PFC [42,44] and serotonergic system in the PFC [44] observed in male and female rat offspring. Other work has reported changes in mRNA expression of the receptor for the metabolic hormones leptin and insulin in the rat hippocampus [29]. Notably, these broad changes in CNS have been observed in rat and mouse models using a range of high fat [29,33,34,40,42], high sucrose [46,47,48] and cafeteria-style [44] diets, and in dams fed diets from pre-mating to lactation (12–14 weeks) [33,40,42], during gestation and lactation (six weeks) [29,34,47], or only during the three weeks of gestation [46] or lactation [44]. 

### 2.2. Affective States

#### 2.2.1. Human Studies

Results from several studies in humans indicate that maternal obesity alters affective states in children. For example, extreme maternal obesity (pre-pregnancy BMI ≥ 40) was associated with hyper-reactivity and affective changes in children, independent of demographic variables, prenatal factors or maternal anxiety and depression [52]. A population-based cohort study, the Western Australian Pregnancy Cohort including 2868 children followed up to 17 years of age, reported that children of mothers with overweight and obesity had higher risk of developing affective problems, independent of demographic and postnatal factors [22]. A longitudinal study of nationwide registries in Finland of 600,000 children followed up to 11 years of age reported increased risk of psychotic, mood and stress-related disorders in children of mothers who were severely obese (pre-pregnancy BMI ≥ 35) [19]. 

#### 2.2.2. Preclinical Studies

By contrast, preclinical studies using affective measures in rodents show mixed results, with maternal obesity found to increase [26,30,31,39,41,53] or decrease anxiety -like behaviour [27,38,43,49]. The age at which behaviour was measured may impact on these findings. While individual studies show mixed results, a meta-analysis examining effects of maternal obesity in rodents (mouse and rat) on anxiety-like behaviour in offspring reported a significant but modest increase in anxiety-like behaviour in offspring from obese relative to control dams [51]. The few studies to assess depression-like behaviour in rodent models of maternal obesity show mixed effects, with increases [30,31] or no differences [41] in immobility in the Forced Swim Test (see Table 2).

Although behavioural measurements differ between studies, preclinical studies have often sought to link affective changes to dysfunctional HPA axis activity, altered neuronal plasticity and increased neuroinflammation. The HPA axis regulates physiological stress responses and modulates behaviour in response to stressful stimuli, and its function is programmed by maternal obesity. An association between hyper- and hypo-active HPA axes and anxiety- and depressive-like behaviour, respectively, has been well documented [54]. Two studies showed that maternal obesity impaired function of the HPA axis feedback system in the hypothalamus, characterised as an increase in corticotrophin releasing factor transcription levels and ACTH response in rat offspring [26] and altered transcription levels of glucocorticoid receptor (Nr3c1) in mouse offspring [27]. Other studies using rats have focused on the hippocampus and amygdala, key regions underlying regulation of emotional states, showing that offspring of obese dams exhibit elevated protein levels and mRNA expression of proinflammatory markers including IL6, TNFα, NFkB and MCP-1 [38,39,41]. Anxiety-like behaviour has been associated with a decrease in serotonin transcription in mice [36], disrupted epigenetic regulation of enzymes in the kynurenine pathway [41] in the rat hippocampus, and altered glutamatergic neurons in the amygdala along with a decrease in epigenetic regulators for neuronal development in mice [53]. Furthermore, changes in epigenetic markers, including Gadd45b and Mecp2, which tightly regulates synthesis of BDNF (brain derived neurotrophic factor) [55,56], have been reported in the prefrontal cortex and amygdala in foetal mouse brain [53]. 

Apart from differences in experimental research protocols (diet used, animal strain, time of test) one possible reason for the discrepancy between the results seen in human research and the mixed results from animal models might relate to methodological differences in reporting negative emotional states in humans and rodents. Human studies tend to use parental reports regarding child emotional states, whereas studies using rodents monitor anxiety- and depressive-like behaviour using ethologically relevant tests in the offspring themselves, such as forced swim, open field, elevated plus maze, dark-light box and sucrose preference tests, which cannot fully capture the breadth of negative affective states in human children. We argue that despite these challenges, the ability to associate behavioural outcomes with neurohumoral and molecular readouts in preclinical research has merit.

### 2.3. Psychosocial Behaviour

#### 2.3.1. Human Studies

The potential link between maternal obesity and Autism Spectrum Disorder (ASD) has been extensively investigated over the past decade or so. Several recent meta-analyses and reviews indicate an increased risk of ASD in children of mothers who were overweight or obese at the time of conception [6,50]. In addition, several studies have examined the impact of maternal obesity on psychosocial development, regulatory behaviour, internalising and externalising behaviour. A prospective cohort study including 3117 mother-child pairs (the PREDO study in Finland) followed up to four years reported that children of obese and overweight mothers had 22% higher odds (95%CI = 5, 42) of developing regulatory behavioural problems (communication, gross motor, fine motor and problem solving) [14]. A longitudinal birth cohort (n = 6850; Early Childhood Longitudinal Study Birth Cohort in USA) found that children of obese mothers were at elevated risk of delayed mental development at two years (RR1.38, 95%CI = 1.03, 1.84) compared with children of mothers with normal BMI [15]. Higher maternal BMI (>25) was a predictor of lower verbal IQ (β = −3.41, 95%CI = −5.69, −1.12), full-scale IQ (β = −3.21, 95%CI = 5.21, −0.88) and externalising behaviour (β = 1.87, 95%CI = 0.72, 3.02) in three–four year-old children in the MIREC-CD study [17], and also predicted externalising behaviour (β = 1.6, 95%CI = 0.45, 2.74) and internalising behaviour (β = 1.2, 95%CI = 0.35, 2.0) at two years of age in the CHILD study [18]. Lastly, several studies using retrospective assessment of dietary patterns by food frequency questionnaires have reported that unhealthy maternal diet during pregnancy was associated with child behavioural problems at three–six years of age [10,11,12,13]. 

#### 2.3.2. Preclinical Studies

Despite growing interest in the link between maternal obesity and aspects of psychosocial behaviour in children in human studies, few studies have explored this relationship in animal models. Research to date has examined social interaction, anxiety and locomotor activity. Several studies have found evidence that neuroinflammation might contribute to impaired social behaviour in offspring of overfed dams: Kang et al. [35] reported that female (but not male) mouse offspring of HFD fed dams exhibited social interaction deficits in the three chamber test if they were fed HFD, but not the control diet. Behavioural changes were accompanied by increased levels of proinflammatory genes, IL1β, TNFα and Iba1 in the brain, and increased glial activity in the amygdala. Maternal western diet consumption decreased diversity of gut microbiota in mouse offspring, and oxytocin expressing neurons in the paraventricular nucleus of the hypothalamus (PVN), and dysregulated DA neurons in the ventral tegmental area (VTA), which was associated with impaired social behaviour in the three chamber test of social interaction [28]. Another study reported that male rat offspring of high fructose diet fed dams displayed impaired sociability which was associated with increased astroglial activity and decreased levels of GABAergic and serotonergic neurotransmitters in the whole brain [45]. In non-human primates, male offspring of mothers fed an energy rich, western style diet showed increased aggressive behaviour [57].

## 3. Underlying Mechanisms by which Maternal Obesity Programs Offspring Behaviour 

Obesity during pregnancy alters neuroendocrine, metabolic and inflammatory status and these exposures might affect foetal development, in turn influencing behaviour, emotion and cognition. In addition to neuroinflammation, altered neuronal plasticity and dysregulated brain metabolism (leptin, insulin and oxytocin) likely contribute. Another mechanism attracting recent attention is changes in the composition of the gut microbiome, which contains trillions of bacteria, and plays a crucial role in health and disease of the host [58]. In the section below, we summarise the relevance of maternal obesity to neurobiological programming in offspring with a focus on roles of microbiota and innate immunity. 

### 3.1. Neuroinflammation 

Rodent models of maternal obesity have shown evidence of hypothalamic inflammation in offspring, characterised by elevated levels of TNFα, IL1b, and IL6 in adulthood, both in rats [59] and mice [60], and excessive proliferation of astrocytes in mouse offspring of obese dams [60]. The hippocampus is also subjected to neuroinflammation induced by maternal obesity, with research in rats showing that hippocampal inflammation is associated with impaired learning in offspring of obese mothers [61]. Cognitive function is closely regulated by physiological levels of proinflammatory cytokines, and aberrant levels of TNFα impair cognitive function in mice [62]. Prolonged neuroinflammation has been linked with behavioural changes and cognitive deficits seen in psychiatric disorders [63]. These hippocampal and hypothalamic changes may underlie cognitive dysfunction in offspring of obese mothers. Thus, exposure to a maternal proinflammatory state during pregnancy and lactation might alter inflammatory homeostasis in the fetal brain and subsequently change brain neuronal circuitries and behaviour. For instance, a study in humans by Rudolph et al. showed that circulating IL6 concentration during pregnancy was associated with newborn brain connectivity and predicted working memory function at two years of age [64]. Neuroinflammation is also implicated in the aetiology of affective and other psychiatric disorders [63]. 

Another possibility is the involvement of glial cells. Glial cells such as microglia and astrocytes are essential for regulating immune responses and act as metabolic sensors [65]. Several studies in mice have shown that consumption of HFD induces glial cell activation, a possible link between dietary lipid consumption and CNS inflammation [66,67,68] in the hypothalamus and the hippocampus. A recent mouse study demonstrated that even a moderate increase in dietary lipids evoked hypothalamic inflammation accompanied by increased hypothalamic GFAP positive cells and microglia, morphological changes in glial cells, altered neuropeptide gene expression levels and elevated proinflammatory gene transcription [69]. In this study, male and female mice were exposed to HFD after brief (2 h) food restriction, and hypothalamic inflammation, neuropeptide gene expressions and glial cell reactivity were measured one, three and six hours later. Notably, strong postprandial hypothalamic inflammation was observed in male mice, whereas the degree of hypothalamic inflammation and neuropeptide gene expression response were weaker in female mice [69]. While sex differences in the inflammatory response to dietary lipids remains to be fully characterised, the exacerbated inflammatory response observed in female mice could manifest as altered immune response in maternal obesity. Thus, glial cells play a key role in the regulation of the inflammatory response to energy dense food intake and may influence feeding behaviour. 

In the context of maternal obesity and effects on offspring behaviour, an experimental study showed changes in glial cell proliferation and reactivity in mouse offspring of obese mothers, characterised by astrocyte proliferation and elevated levels of IL6 in the hypothalamic arcuate nucleus (ARC) and the supraoptic nucleus (SON) compared with offspring from normal weight dams [60]. This evidence demonstrates how prenatal exposure to energy dense food and metabolic syndrome conferred by maternal obesity can disrupt glial populations in offspring, potentially driving the onset of low-grade inflammation. Intriguing new evidence indicates that specific manipulations of nutrient content of the mother’s diet can alter microglial function. Madore et al. [70] showed that offspring of mouse dams fed a diet deficient in n-3 fatty acids exhibited cognitive impairment (Y-maze and recognition memory tests) and disrupted microglial morphology in the CA1 region of hippocampus. 

Lastly, increasing evidence suggests an intriguing relationship between glial cells and the gut microbiome. Preclinical studies using conventional and germ-free mice showed that the gut microbiota indirectly regulate homeostasis and immune reactivity of microglia [71]. Gut microbiome composition also indirectly regulates astrocyte function: a study using an animal model of multiple sclerosis showed that dietary tryptophan metabolites from *Lactobacillus reuteri* suppressed neuroinflammation by binding to a receptor expressed on astrocytes [72]. Neuroinflammation has been implicated in the dysregulation of glutamate clearance by glial and neuronal cells, which could subsequently contribute to abnormal behaviour [73]. Notably, *L.reuteri* produces gamma-aminobutyric acid (GABA) which has inhibitory effects on neuronal circuity [74]. A recent study demonstrated a possible mechanism whereby *L.reuteri* regulates social behaviour in mice via the vagus nerve pathway interacting with oxytocin in the VTA [75]. In summary, maternal obesity-induced bioactive proinflammatory compounds can be transferred to offspring during gestation and lactation, which could have negative influences on offspring neurodevelopment and immune profile through an interaction between glial cells and gut microbiome. 

### 3.2. Altered Brain Development: Structure, Connectivity and Neurotransmitter Function 

Studies in rodents and humans link maternal obesity to neurological changes in offspring. A recent human study reported a negative association between maternal BMI and hippocampal volume in male children at 7–11 years of age [76]. Furthermore, consumption of diets high in polyunsaturated omega-6 fatty acids during pregnancy impairs healthy brain structure and development in the foetus. Exposure to high omega-6 fatty acid concentrations in utero desensitises cortical neurons in the mouse foetus brain, affecting neuronal differentiation, which is essential for development of normal brain structure [77]. Maternal high fat diet consumption affects POMC and NPY mRNA expression in the arcuate nucleus (ARC) of the hypothalamus, a critical region for regulating feeding and energy balance. Preclinical studies indicate that levels of POMC and NPY mRNA expression fluctuate across the developing rat brain, with POMC and NPY mRNA expression in the ARC found to be increased on embryonic day 18 [78], while POMC is increased and NPY decreased at postnatal day 21 [79]. A recent study showed that higher plasma maternal IL-6 levels during the third trimester were associated with elevated anxiety-like behaviour in non-human primate female offspring at 11 months of age via differences in the amygdala volume at four months of age [80]. Although the focus of the study was not maternal obesity, and hence mothers were not obese, this intriguing finding that the relationship between maternal IL-6 levels and anxiety-like behaviour was mediated by the development of amygdala in female offspring implicates potential adverse effects of maternal obesity on offspring brain development. Reductions in the epigenetic factors DNA methylase (DNMT1) at PND1 and histone deacetylase, SIRT1 and HDAC1, have been observed in the ARC in offspring from HFD fed dams [81], with these changes accompanied by hyperphagia. These CNS structural and functional changes in offspring of obese mothers can potentially dysregulate brain circuities, and more work is needed to characterise these longitudinally. 

### 3.3. Brain Connectivity and Neuronal Circuits

Recent studies using functional magnetic resonance imaging technology indicate an association between higher pre-pregnancy BMI and altered white matter microstructure [24], neuronal circuitry and connectivity [21,23] in the child’s brain. Multiple studies have reported hypo- or hyper-connectivity between hemispheres in children of obese mothers. While maternal obesity studies have often focused on the hippocampus and hypothalamus, several studies now support a critical role for the striatum, through its role in integrating information from the frontal cortex, temporal cortex and amygdala to guide behaviour [82,83,84]. The VTA and habenula regulate aspects of motivation and decision making [85]. Therefore, future investigations on the effects of maternal obesity on behaviour in animal models should consider integrated neuronal circuitries rather than region specific changes. 

### 3.4. Dopaminergic and Serotonergic Systems

Dopamine is a neurotransmitter regulating motivational and reward-related functions. A recent preclinical study showed that consumption of cafeteria diets during pregnancy induced epigenetic changes in the dopamine receptor (DAT), dopamine transporter 1 (DRD1) and dopamine transporter 2 (DRD2) genes in the nucleus accumbens (NAc) and VTA, two key regions of the dopaminergic pathway [86]. In the VTA and NAc, DRD1, DRD2 and DAT expression was decreased at embryonic day 2 and postnatal day 10 in rat female offspring. Similarly, dopaminergic neuronal circuits in mouse offspring were altered by maternal high fat diet consumption during lactation, resulting in hyperlocomotion in male offspring and increased palatable food and sucrose intake in female offspring [87]. Intriguingly, the timing of maternal overnutrition and maternal dietary pattern with respect to pregnancy can also influence neural circuitries in offspring. For instance, Sarker el al. [88] showed maternal HFD consumption early in gestation was associated with lower DA levels in adult male mouse offspring and higher DA levels in adult female mouse offspring in the NAc compared to their respective controls. Lower DA levels in the NAc were found in male and female offspring when dams were fed HFD later in gestation. Recent RNA sequencing work in the frontal cortex of rat offspring from dams fed HFD during gestation and lactation suggests that there may be changes in the balance between excitatory and inhibitory neuronal cell markers compared to offspring from dams fed standard diets [89]. These studies indicate programming effects of maternal obesity on neurochemical development in the offspring brain. From a translational perspective, we need to apply caution when extrapolating these findings from rodents to human neurodevelopment due to differential brain development across species.

Perinatal exposure to maternal obesity and poor diet induces lasting effects on the central serotonergic system in female offspring [57]. High fat diet consumption during pregnancy increased anxiety-like behaviour and cortisol levels in non-human primate offspring, which was associated with lower tryptophan hydroxylase-2 (TPH2) mRNA expression in the dorsal and median raphe and altered 5HT immunoreactivity in the prefrontal cortex in juvenile non-human primates [90]. TPH2 is an essential enzyme in the production of central 5HT and the dysregulation of this enzyme has been implicated in altered stress responses [91]. Interestingly, the pro-inflammatory cytokine TNFα contributes to depressive-like behaviour by modifying the serotonergic system [92]. 

### 3.5. Roles of Metabolic Hormones in the Brain 

Evidence from animal models shows maternal obesity programs the rat offspring’s health trajectory towards obesity by impaired lipid and glucose metabolism and appetite regulators [93,94]. Interestingly, in a rat model of maternal obesity, Morris and Chen found significantly lower plasma leptin levels in postnatal day 1 rat offspring, along with lower levels of hypothalamic mRNA expression of NPY, leptin receptor and STAT3 [95]. The lowered plasma leptin levels at day 1 were later normalised and increased at day 10. How these early changes in plasma leptin affect offspring later in life requires further investigation. Furthermore, Park et al. [96] showed maternal obesity resulted in postnatal endoplasmic reticulum (ER) stress in the pancreas and hypothalamus in mouse offspring. Maternal obesity in this mouse model also disrupted the development of melanocortin circuits associated with neonatal hyperleptinemia and leptin resistance in the mouse hypothalamus [96]. Similarly, maternal obesity programs neuronal insulin resistance in the mouse hippocampus [97]. While studies of brain insulin levels have focused on the hypothalamus, given its role in appetite control, insulin plays a critical role in CNS regulation by controlling proliferation of neuronal stem cells, structural plasticity and synaptic plasticity in the hippocampus, which is essential for healthy cognitive functioning [98]. 

Maternal overnutrition also alters the endocannabinoid system through impaired leptin signalling in the hypothalamus. Interestingly, only male rat offspring of HFD-fed dams exhibited impaired leptin signalling, with epigenetic changes in promoter regions of sex hormone signalling at birth [99]. The sex-specific changes could amplify effects of maternal obesity on neurodevelopment later in life. Leptin exerts protective and neurotrophic effects and hence its potential use as an antidepressant has been proposed [100]; however, the current evidence for therapeutic effects of leptin is inconsistent [101]. Notably, in humans, higher leptin concentrations in breast milk were found in obese/overweight mothers compared to normal weight mothers [102]. While milk leptin levels decreased across lactation in normal weight mothers, the association was absent in obese/overweight mothers, where milk leptin remained high across lactation. Although there was no association between milk leptin levels in obese mothers and infant body weight in the study, the higher leptin concentration during lactation may have long lasting impacts on neurodevelopment in offspring. A recent human study reported maternal obesity affected levels of mRNA expression and DNA methylation of the leptin and adiponectin systems in placenta during the third trimester [103]. The study did not follow up the child’s emotional state; longitudinal studies could provide insight into how maternal obesity could affect leptin levels in offspring and associated behavioural changes. 

The adverse changes in brain metabolism in offspring from obese dams include altered mammalian target of rapamycin (mTOR) signalling in the rat hypothalamus [79] and in the mouse hippocampus [97]. Dysregulation of mTOR signalling has been implicated in memory formation [104,105], developmental disorders such as autism spectrum disorder [106,107] and mood disorders [108]. mTOR signalling and proinflammatory mediators such as IL1β and TNFα have a reciprocal relationship and together maintain innate immune homeostasis [109,110]. As mentioned above, maternal obesity promotes a proinflammatory state in the periphery and CNS, and proinflammatory mediators could transfer to offspring via placenta and breast milk. Such inflammatory mediators interact with insulin, leading to insulin insensitivity and hypercortisolemia, can contribute to desensitisation of glucocorticoid receptors; all of these have been implicated in the aetiology of depression [111]. In humans, maternal obesity and metabolic status programs cortisol reactivity at three–five years of age [112]. Higher maternal pre-pregnancy BMI is associated with lower levels of salivary cortisol in young adult offspring [113], independent of the child’s BMI.

Oxytocin is another brain hormone for which there is evidence of a relationship between maternal obesity and aberrant offspring behaviour. Oxytocinergic neurons in the PVN project to the amygdala, hippocampus, NAc and VTA, and peripherally project to the pituitary into the blood stream [114,115]. Oxytocin is known to regulate homeostatic, reward and impulse behaviour in relation to eating behaviour in humans [114]. As PVN oxytocin neurons express insulin receptors, projections of oxytocinergic neurons are regulated by central insulin [116]. Preclinical evidence shows that maternal obesity induces metabolic alterations and abnormal hypothalamic development in mouse [96] and rat [59,117] offspring, suggesting that central oxytocin levels in these offspring might be affected. Oxytocin plays a key role in social cognition. For instance, central oxytocin injection modulates maternal obesity-induced impairments in social behaviour in male mouse offspring via DA neurons in the VTA [28]. Furthermore, male mouse offspring of obese dams showed an increase in oxytocin receptor mRNA expression with epigenetic changes in the hippocampus at late gestational stage [32]. Hence oxytocin has influence on both brain circuitries and regulation of metabolic status, both of which may underlie behavioural changes induced by maternal obesity. 

### 3.6. Gut Microbiota Composition

The ‘gut-brain axis’ describes the complex communication network between the gut and brain, comprising the enteric nervous system, sympathetic and parasympathetic branches of the autonomic nervous system, in addition to neuroendocrine signalling pathways, and neuroimmune systems [118]. Emerging experimental and clinical evidence supports a role for the gut microbiome-brain axis in influencing mammalian behaviour. There is increasing recognition that the gut microbiome shapes neurological development and function [119,120], and potentially contributes to neurodevelopment and behaviour in offspring. Relevant literature has been reviewed elsewhere [121,122]; here, we have briefly summarised relevant studies.

Only limited evidence is currently available for an association between maternal diet and offspring gut microbiome composition in humans. Savage et al. showed a weak but positive association between maternal diet, characterised by high vegetable intake and low processed/fried food intake, and *lactobacillus* spp. abundance in the infant gut [123]. A recent study demonstrated that germ-free mice colonised with microbiota from infants of obese mothers developed signs of non-alcoholic fatty liver disease (NAFLD) including increased hepatic gene expression for endoplasmic reticulum stress and innate immunity, compared to mice colonised with faeces from human infants of normal weight mothers [124]. Exposing these mice to western style diets accelerated weight gain and the onset of NAFLD [124]. A caveat for this result is that germ-free animals differ from standard pathogen free animals in many aspects of innate immune system and neurodevelopment [125]. We have shown that the faecal microbiome of offspring is strongly modulated by maternal diet, with reductions in diversity in both mothers consuming HFD, as well as their offspring [126]. Therefore, more longitudinal studies are needed to investigate how maternal obesity affects the gut microbiome, behaviour and neurodevelopment in offspring across their development. Given that the gut microbial community is a dynamic and complex system which is sensitive to factors such as living conditions, lifestyle and geography, investigations using animal models are useful to identify the precise effects of macronutrient, micronutrient, prebiotics and probiotics on the gut microbial community in offspring and any impact on behaviour. Also, investigations on how maternal diets impacts on time course of offspring gut microbiota development will provide important insights. It is noteworthy that host health and environmental factors modulate the effects of supplementation with probiotics on the host gut microbiome, not necessarily resulting in the same gut microbial community [127].

Lactation is another key period that shapes the offspring’s gut microbiome, immune system and behaviour. Breast milk is comprised of non-nutrient components such as immunoglobulins, oligosaccharides, growth factors, and epithelial and immune cells, which could reflect maternal health, and DNA—essential bioactive compounds that foster the development of the gastrointestinal, immune and neurological systems in offspring [128]. Indeed, the bacterial communities found in maternal milk correlate with micronutrient profiles in maternal diet. Studies have demonstrated that maternal intake of fatty acids, carbohydrates and protein were associated with the relative abundance of several taxa in human breast milk [129,130]. Another study showed that obese mothers exhibited higher total bacteria counts, higher numbers of *Staphylococcus* and *Lactobacillus* and lower numbers of *Bifidobacterium* bacteria relative to normal weight mothers [131]. A recent study examining breast milk from 393 mother-infant dyads showed that maternal BMI directly influenced breast milk components such as insulin, lipids and cytokines and further indirectly modulated bacteria in the breast milk [132]. What is still unknown is to what degree breast milk components interact with other maternal factors, such as diet, to influence gut microbiome and neurodevelopment in offspring. Hence, further investigations using animal studies are crucial to determine the factors associated with the process of maturation of the infant gut microbiome.

## 4. Conclusions

There is now broad evidence from human and animal studies indicating that maternal obesity, overnutrition and unhealthy dietary patterns induce adverse effects on behaviour and neurodevelopment in offspring. This review has identified possible underlying neurobiological mechanisms for these effects, which include neuroinflammation, disrupted neural circuitries and connectivity and dysregulated brain hormones. Dynamic interactions between these factors shape behaviour during gestation and lactation through the offspring’s exposure to maternal obesity via the placenta, gut microbiome and breast milk. Future research should continue to examine behaviour and brain changes in maternal obesity models beyond the hippocampus and hypothalamus, given recent evidence from imaging studies suggesting that aberrant behaviour might be a result of dysregulated global network in the brain, in addition to region-specific dysfunctions. In addition, future research should be targeted at characterising the intriguing sex differences evident in many maternal obesity studies, given that current preclinical studies are still skewed towards males. Human studies using advanced imaging techniques could provide a useful resource to guide animal studies to further elaborate underlying mechanisms. Further, animal models need to incorporate experimental protocols that maximise translational relevance to align with human studies in terms of dietary components, feeding duration and behavioural tests, to allow behavioural and molecular outcomes to be systematically assessed longitudinally and compared across species where possible. Studies currently underway should yield more valuable data on the longer-term effects in humans.

## Figures and Tables

**Table 1:** Summary of clinical studies examining associations between maternal obesity and diet quality on offspring brain development and behaviour (2010–2020).

	Cohort/Study Aim	Child Age	Main Findings
**Study Type: Maternal Dietary Pattern on Child Behaviour**			
Cohen et al. [10]	1234 mother-child pairs enrolled 1999–2002 in Project Viva Maternal measures: pre-pregnancy sugar consumption Outcomes in children: sugar consumption and cognition	Early and mid-childhood (median ages 3.3 and 7.7 years)	Higher maternal sucrose consumption (mean 49.8 g/day [SD12.9] was associated with lower mild-childhood KBIT-II (−1.5 points per 15 grams/day, 95%CI = −2.8, −0.2) at 7.7 years of age, and with lower KBIT-II verbal scores (−1.5, 95%CI = −2.4, 0.0) and non-verbal scores (−3.2 95%CI = −5.0, −1.5) in children at 7.7 years. Verbal score association remained after adjusting for multiple comparisons.
Galera et al. [11]	1242 mother-child pairs from a French cohort, EDEN mother-child cohort Maternal measures: dietary patterns during pregnancy Outcomes in children: hyperactivity-inattention and conduct problems (externalising behaviour)	3 to 8 years	Maternal ‘low Healthy (high in vegetables, fruits, nuts, and seafood) diet’ (adjusted Odds Ratio (aOR) = 1.61, 95%CI = 1.09–2.37) and ‘high Western (highly processed foods, high in carbohydrates, saturated and trans fats) diet’ (aOR = 1.67, 95%CI = 1.13–2.47) during pregnancy associated with children’s trajectories of high symptoms of hyperactivity-inattention but not with conduct problems.
Jacka et al. [12]	23,020 mothers and their children enrolled in Norwegian Mother and Child Cohort Study Maternal measures: Dietary patterns during pregnancy Outcomes in children: internalising and externalising problems	1.5, 3 and 5 years	Higher intakes of ‘unhealthy’ dietary pattern (high intake of processed meat products, refined cereals, sweet drinks and salty snacks) during pregnancy predicted externalising problems in CBCL among children from 1.5 to 5 years of age. This relationship was not confounded by other factors, nor explained by children’s postnatal diets.
Steenweg-de Graaf et al. [13]	3062 mothers/3104 children enrolled in the Generation R Study Cohort Maternal measures: dietary pattern during pregnancy Outcomes in children: internalising and externalising problems	1.5, 3 and 6 years	High adherence to Mediterranean dietary pattern (high vegetables, fish & shellfish, vegetable oil, fruit and eggs) during pregnancy was negatively associated (OR 0.9, 95%CI = 0.83, 0.97) and high adherence to the Traditional Dutch dietary pattern (high intake of fresh and processed meat and potatoes, a relatively high intake of margarines and a very low intake of soy and diet products) was positively associated (OR 1.11, 95%CI = 1.03,1.21) with child externalising problems after adjusting for pre-pregnancy BMI, average daily caloric intake during pregnancy, and child consumption of snacks and sugar containing beverages at 3–6 years.
**Study type: Maternal obesity on child behaviour**			
Girchenko et al. [14]	3117 mother-child pairs from PREDO study Maternal measures: pre-pregnancy BMI and comorbid disorders (hypertensive disorders and gestational diabetes) Outcomes in children: regulatory behaviour problems (communication, gross motor, fine motor, problem solving, and personal/social domains of development)	16.9 days and 42.2 months	Children of overweight/obese mothers had higher levels of regulatory behaviour problems; 22% higher odds (95%CI = 5–42%) of problems on multiple domains of behavioural regulation, mean age 16.9 days compared to offspring of normal weight mothers. Infant regulatory behaviour problems partially mediated the association between maternal overweight/obesity and developmental milestones (communication, gross motor, fine motor, problem solving and personal/social domains of development).
Hinkle et al. [15]	Early Childhood Longitudinal Study-Birth Cohort (n = 6850) Maternal measures: pre-pregnancy BMI Outcomes in children: mental development index (MDI) and psychomotor development index (PDI)	2 years	Mental development index of children of underweight (BMI < 18.5), overweight (BMI = 25.0–29.9), obese class I (BMI 30.0–34.9) and obese class II and III (BMI ≥ 35.0) mothers lower compared to children of normal weight mothers (−2.13, 95%CI = −3.32, −0.93). Adjusted risk of delayed mental development was increased in children of class II and III obese mothers (RR 1.38, 95%CI = 1.03, 1.84). Children’s psychomotor development index did not associate with maternal pre-pregnancy BMI.
Jo et al. [16]	1311 mother-child pairs enrolled in the Infant Feeding Practices Study II Maternal measures: pre-pregnancy BMI Outcomes in children: psychosocial development	6 years	Children of obese class II & III mothers (BMI ≥ 35.0) had increased odds of emotional symptoms (aOR 2.24, 95%CI = 1.27, 3.98), peer problems (aOR 2.07, 95%CI = 1.26,3.24), total psychosocial difficulties (aOR 2.17, 95%CI = 1.24, 3.77), attention-deficit/hyperactivity disorder diagnosis (aOR 4.55, 95%CI = 1.80, 11.46), autism or development delay diagnosis (aOR 3.13, 95%CI = 1.10–8.94), receipt of speech language therapy (aOR 1.93, 95%CI = 1.18, 3.15), receipt of psychological service (aOR 2.27, 95%CI = 1.09, 4.73), and receipt of any special needs service (aOR 1.99, 95%CI = 1.33–2.97) compared with children of normal weight mothers (BMI 18.5–24.9). Results held after adjustment for pregnancy weight gain, gestational diabetes, breastfeeding duration, postpartum depression and child’s birth weight.
Krzeczkowski et al. [17]	808 mother-child pairs from the Maternal Infant Research on Environmental Chemicals-Child Development Plus (MIREC-CD plus) cohort Maternal measures: pre-pregnancy BMI and hyperglycaemia Outcomes in children: verbal, performance and full-scale IQ, internalising and externalising problems	3–4 years	In the unadjusted models, higher BMI (>25) predicts lower verbal IQ (β = −3.41, 95%CI = −5.69, −1.12), Full-scale IQ (β = −3.21, 95%CI = −5.21, −0.88) and Externalising (β = 1.87, 95%CI = 0.72, 3.02). Associations were not significant after adjustment for prenatal diet, home environment, maternal depression, education and prenatal smoking. Post-hoc semi-partial correlations indicated prenatal diet and home environment accounted for significant variance in verbal and full-scale IQ.
Krzeczkowski et al. [18]	815 mother-child pairs enrolled at the Edmonton site of the Canadian Healthy Infant Longitudinal Development (the CHILD study) cohort Maternal measures: pre-pregnancy BMI, GDM and GWG Outcomes in children: externalising and internalising problems	2 years	In the unadjusted models, higher BMI (>25) predicted externalising (β = 1.6, 95%CI = 0.45, 2.74) and internalising (β = 1.2, 95%CI = 0.35, 2.0) problems in children. Results not significant after adjusting for gestational diet, socioeconomic status, postpartum depression, prenatal smoking and breastfeeding. Post-hoc analyses indicated that gestational diet accounted for significant variance in both externalising (semi-partial r_diet_ = −0.20, *p* < 0.001) and internalising (semi-partial r_diet_ = −0.16, *p* = 0.01) problems.
Kong et al. [19]	Nationwide national registries in Finland between 2004 and 2014 (n = 649043) Maternal measures: maternal obesity (pre-pregnancy BMI), pregestational diabetes mellites and gestational diabetes mellitus Outcomes in children: risk of neurodevelopmental disorder	Up to 11 years	Severely obese mothers without diabetes (pre-pregnancy BMI ≥ 35) had 67% to 88% increased risk of child having mild neuro-developmental disorders (hazard risk ratio (HR) = 1.69, 95%CI = 1.54, 1.86), attention-deficit/hyperactivity disorder or conduct disorder (HR = 1.88, 95%CI = 1.58, 2.23) and psychotic, mood, and stress-related disorders (HR = 1.67, 95%CI = 1.31, 2.13) compared with mothers with normal BMI.
Monthe-Dreze et al. [20]	1361 mother-child pairs enrolled 1999–2002 in Project Viva Maternal measures: pre-pregnancy BMI Outcomes in children: cognition	Early and mid-childhood (median ages 3.3 and 7.7 years)	Children of mothers with high pre-pregnancy BMI (≥30) had lower WRAVMA (Wide Range Assessment of Visual Motor Abilities) (β = −2.1, 95%CI = −3.9, −0.2) at 3.3 years compared with children of mothers with normal BMI. Association was attenuated after adjusting maternal CRP (β = 1.8, 95%CI = −3.8, 0.2) but not other inflammatory markers including n−6:n−3 PUFA ratio and Dietary inflammatory index. Pre-pregnancy BMI was not associated with child cognitive outcomes at 7.7 years.
Norr et al. [21]	109 pregnant women from a community sample. Maternal measures: pre-pregnancy BMI Outcomes in children: macrocircuitry and connectivity in fetal brain by fMRI	26 to 39 weeks gestational age	Strength of connectivity between two subnetworks, left anterior insula/inferior frontal gyrus and bilateral prefrontal cortex, varied with maternal BMI. In prefrontal and left insular cortical regions, both increased and decreased between-network connectivity with a tendency for increased within-hemisphere connectivity and decreased cross-hemisphere connectivity in higher BMI pregnancies.
Robinson et al. [22]	2900 mothers and 2868 children in the Western Australian Pregnancy Cohort (Raine) study Maternal measures: pre-pregnancy BMI Outcomes in children: affective problems (dysthymic disorder and major depressive disorder)	5 to 17 years	Higher risk of affective problems (measured by CBCL/DSM-IV) between 5 and 17 years among children of women who were overweight (pre-pregnancy BMI ≥ 25.0: OR 1.51 95%CI = 1.08, 2.12) and obese (pre-pregnancy BMI ≥ 30.0: OR 1.72 95%CI = 1.11, 2.67) after adjustment for confounders, including maternal age, maternal education, stress in pregnancy, gestational age at birth, birth weight, gestational diabetes and length of breastfeeding.
Shapiro et al. [23]	101 mother-child pairs enrolled in the Healthy Start study, a longitudinal prebirth cohort Maternal measures: pre-pregnancy BMI Outcomes in children: brain connectivity and neuronal activity by rs-fMRI	4–6 years	Children of mothers with overweight or obesity had hyperactivity in the left posterior cingulate cortex and hypoactivity in the left anterior prefrontal cortex the dorsal anterior cingulate and supplementary motor area at 4–6 years, independent of child age, sex and BMI, maternal smoking and education. After adjusting for child BMI, sex, and maternal smoking, children born to overweight/obese mothers had weaker brain connectivity between regions involved in cognitive control and salience.
Verdejo-Roman et al. [24]	Data from three European birth cohorts; children at 6 years of age (n = 116) from the PREOBE study; children at 10 years of age (n = 2466) from the Generation R study; and young adult at 26 years of age (n = 437) from the NFBC 1986 study. Maternal measures: pre-pregnancy BMI Outcomes in children: white matter microstructure by MRI	6, 10 and 26 years	After adjusting for education, smoking and alcohol use, maternal BMI was associated with higher FA and lower MD in multiple brain tracts (i.e., association and projection fibres) in children aged 10 and 26 years, but not at 6 years. None of the associations of maternal BMI with individual white matter tracts were consistent across the two cohorts.
Widen et al. [25]	2084 Mother-child pairs enrolled in the Child Health and Development Studies. Maternal measures: pre-pregnancy BMI and GWG Outcomes in children: cognition	9 years	Pre-pregnancy overweight (BMI 25–29.9) and obesity (BMI ≥ 30) were associated with lower Peabody scores (Peabody Picture Vocabulary Test; assess receptive vocabulary) in mid-childhood (β = −1.29, 95%CI = −2.6,−0.04) and (β = −2.7, 95%CI = −5.0, −0.32) respectively. GWG was not associated with child Peabody score. Maternal pre-pregnancy BMI and GWG were not associated with child Raven score (Raven Coloured Progressive Matrices Tests; assess perceptual reasoning and cognitive functioning) at 9 years of age.

KBIT-II, Kaufman Brief Intelligence Test; BMI, body mass index; CBCL, Child Behaviour Checklist; aOR, adjusted odds ratio; OR, odds ratio; GWG, gestational weight gain; GDM, gestational diabetes mellitus; FA, Magnetic resonance imaging-derived fractional anisotropy; MD, mean diffusivity.

**Table 2:** Summary of preclinical studies examining effects of maternal overnutrition on offspring brain development and behaviour (2010–2020).

Study	Species	Maternal Diet	Maternal Diet Duration (Total in Weeks)	Offspring Sex	Brain Regions	Effect of Maternal Overnutrition on Offspring Behavioural and Neural Outcomes
**Types of maternal overnutrition—high fat diet or cafeteria diet (offspring was weaned on CD)**						
Abuaish et al. [26]	Rat, Long Evans	HFD (20% protein, 60% fat, 20% carbohydrate) CD (28.5% protein, 13.5% fat, and 58% carbohydrate)	Mating Gestation Lactation (8 weeks)	Male Female	Hypothalamus PVN Hippocampus	Target behaviour: Anxiety-like behaviour @PND7 Increased anxiety-like behaviour during the stress hyporesponsive period, characterised by ↑USV, ↑ immobility in presence of threatening stimuli, ↑ *Crh* mRNA expression in PVN; ↑ACTH response. Impaired HPA axis feedback as shown by lower Nr3c1 mRNA levels in ventral hippocampus at PND13. No sex difference in any measures.
Balsevich et al. [27]	Mouse, C57BL6	HFD (17% protein, 58% fat, 25% carbohydrate) CD (17% protein, 11% fat, 73% carbohydrate)	Mating Gestation Lactation (9 weeks)	Male	Hypothalamus PVN, ARC, MVH	Target behaviour: Anxiety-like behaviour @3/12 months Young adult (3 months) showed less anxious-like behaviour characterised by ↑ distance travelled in OFT, ↑time spent in open arm and open arm entry in EPM and ↑ latency to float in FST, which was associated with ↓Nr3c1mRNA expression in ARC, VMH and ↑Fkbp5 mRNA expression in PVN Adult (12 months) showed anxious-like behaviour characterised by ↓time spent in open arm in EPM, ↑time floating and latency to float in FST, which was associated with ↑Nr3c1mRNA expression in PVN, ARC and VMH and ↑Fkbp5mRNA expression in PVN and ARC
Buffington et al. [28]	Mouse, C57BL6/J	HFD (20% protein, 60% fat, 20% carbohydrate) CD (30% protein, 13.4% fat, 57% carbohydrate)	Pre-mating Gestation Lactation (14 weeks)	Male	Hypothalamus PVN VTA	Target behaviour: social behaviour @7–12 weeks old Impaired social behaviour by reduced interaction time with other mice (a familiar or novel mouse) in the three-chamber test. Reduced oxytocin expressing neurons in the PVN. Failed to induce synaptic potentiation in the VTA DA neurons in response to social interaction. Co-housing with offspring of CD dams reversed impaired social behaviour and dysbiosis in gut microbiome in offspring of HFD dams. Treatment with a bacterium, L. reuteri or Oxytocin reversed impaired synaptic potentiation in the VTA DA neurons and social behaviour.
Cordner et al. [29]	Rat, Sprague Dawley	HFD (20% protein, 60% fat, 20% carbohydrate) CD (28.5% protein, 13.5% fat, 58% carbohydrate)	Gestation Lactation (6 weeks)	Male	Hippocampus	Target behaviour: cognition @PND95 Impaired cognitive impairment by reduced time to explore a novel object in NOR. ↓ Insr, Lepr, and Slc2a1 (GLUT1) mRNA expression in hippocampus at PND21. ↓Insr and Lepr persisted at PND150.
Gawlinska et al. [30]	Rat, Wistar	HFD (60% fat) CD (13% fat) Other macronutrient components unknown.	Gestation Lactation (6 weeks)	Male Female	Hippocampus	Target behaviour: Depressive-like behaviour @PND28/63 Depressive-like behaviour by ↑immobility in FST at both stages of life. The behavioural change was associated with ↑ serum and hippocampal irisin levels at PND28 and ↓IL1α protein levels in the hippocampus at PND28 and PND63 in female offspring.
Giriko et al. [31]	Rat, Wistar	HFD (27.1% protein, 52.0% fat, 20.9% carbohydrate) CD (33.0% protein, 14.7% fat, 52.2% carbohydrate)	Lactation (3 weeks)	Male	n/a	Target behaviour: Aggressive, Depressive-like behaviour @PND60/90 Depressive-like behaviour shown by ↓swimming and climbing in FST at PND60, ↑ aggressiveness in foot shock test at PND110. Other notable physical changes; delays in head growth, delays in the maturation of physical features, reflex ontogenies and ↑ weight gain.
Glendining et al. [32]	Mouse, C57BL/6	HFD (20% protein, 45% fat, 35% carbohydrate) CD (20% protein, 10% fat, 70% carbohydrate)	Pre-mating Mating Gestation Lactation (14 weeks)	Male Female	Mediobasal hypothalamus Amygdala Hippocampus Medial PFC	Target behaviour: Anxiety-like behaviour @PND8/21 ↑anxiety-like behaviour characterised as altered USV at PND8 and adult female offspring (PND21) by the EPM. Altered epigenetic regulations at gestational day 17.5, ↓Gadd45b mRNA expression in the medial PFC, ↓ Mecp2 mRNA expression in Amygdala. ↓ Crebbp, Dnmt3b, and Mecp2 in male hippocampus. At PND8, ↑ glutamatergic neurons in the basomedial nucleus in amygdala
Graf et al. [33]	Mouse, C57BL/6	HFD (60% fat) CD (10% fat) Other macronutrient components unknown.	Pre-mating Mating Gestation Lactation (14 weeks)	Male Female	Whole brain	Target behaviour: Cognition @16 weeks old Male offspring showed cognitive impairment (less interest in novel object in NOR). At PND0, ↑IL1β, TNFα, Nurr77 and Nurr1 mRNA expression in whole brain homogenates; ↓myelination in male medial cortex at PND21.
Janthakhin et al. [34]	Rat, Wistar	HFD (20% protein, 45% fat, 35% carbohydrates) CD (20% protein, 10% fat, 70% carbohydrate)	Gestation Lactation (6 weeks)	Male	Hippocampus Amygdala	Target behaviour: Cognition @3/5 months old Memory impairment in the conditioned odour aversion test behavioural change was associated with ↑dendric complexity of pyramidal neurons in the basolateral amygdala and hippocampus CA1 regions.
Kang et al. [35]	Mouse C57BL/6J	HFD (20% protein, 60% fat, 20% carbohydrate) CD (20% protein, 10% fat, 70% carbohydrate)	Mating Gestation Lactation (12 weeks)	Male Female	Hemi-brain Amygdala	Target behaviour: Autistic-like behaviour @PND32–35 Female offspring showed increased anxiety-like behaviour in OFT while Male offspring showed anxiolytic behaviour in OFT. Female offspring showed ↑ social deficit in three chamber test. Behavioural change was associated with ↑ Iba1, TNFα and IL1β mRNA in hemi-brain and ↑reactive Iba1 stain in the amygdala in female offspring.
Peleg-Raibstein et al. [36]	Mouse C57BL/6N	HFD (60% fat) CD (unknown) Other macronutrient components unknown.	Pre-mating Mating Gestation Lactation (9 weeks)	Male Female	Dorsal and Ventral Hippocampus	Target behaviour: Anxiety-like behaviour @PND90 ↑anxiety-like behaviour on the EPM. ↑ latency to consume novel food. ↑BDNF mRNA in the dorsal hippocampus; ↑ 5-HT1A and GABAa2 mRNA in the ventral hippocampus. No sex differences.
Robb et al. [37]	Rat Sprague Dawley	HFD (20% protein, 45% fat, 35% carbohydrate) CD (20% protein, 10% fat, 70% carbohydrate)	Pre-mating Mating Gestation Lactation (18 weeks)	Male Female	Hippocampus	Target behaviour: spatial memory @PND40/90 Impaired spatial memory in male offspring by ↓MWM acquisition at PND40 (late adolescence). No offspring brain data in offspring was provided.
Sasaki et al. [38]	Rat, Long Evans	HFD (20% protein, 60% fat, 20% carbohydrate) CD (28.5% protein, 13.5% fat, 58% carbohydrates)	Pre-mating Mating Gestation Lactation (10 weeks)	Male Female	Hippocampus Amygdala	Target behaviour: Anxiety-like behaviour @PND45 Decreased anxiety-like behaviour in EPM, OFT and Light-Dark box, which associated with ↑GR, NFkB, IL6, IkBa, and MKP-1 mRNA expression in the hippocampus, and ↑IL-1Ra mRNA expression in the amygdala.
Sasaki et al. [39]	Rat, Long Evans	HFD (20% protein, 60% fat, 20% carbohydrate) CD (28.5% protein, 13.5% fat, 58% carbohydrate)	Pre-mating Mating Gestation Lactation (10 weeks)	Male Female	Hippocampus Amygdala	Target behaviour: Anxiety-like behaviour @PND90 ↑anxiety-like behaviour in Light-Dark box, OFT and EPM, which associated with↑ MR, GR, NFkB, and IL6 mRNA expression in the amygdala; ↓IkBa and IL1Ra mRNA expression in the hippocampus; and ↓IL-1Ra mRNA levels in the amygdala.
Tozuka et al. [40]	Mouse C57BL/6J	HFD (25% protein, 32.4% fat, 28.8% carbohydrate) CD (25.6% protein, 4.0% fat, 50.5 carbohydrate)	Pre-mating Mating Gestation Lactation (12 weeks)	Male	Hippocampus	Target behaviour: Learning and memory @3/4/9/10 weeks old ↓ spatial learning in Barnes maze at 3/4 weeks old was associated with ↓ dendric arborization of new hippocampal neurons and ↓ hippocampal BDNA mRNA expression and protein level.
Winther et al. [41]	Rat, Sprague Dawley	HFD (20% protein, 60% fat, 20% carbohydrate) CD (20% protein, 10% fat, 70% carbohydrate)	Pre-mating Mating Gestation Lactation (14 weeks)	Male Female	Hippocampus	Target behaviour: Anxiety- and Depressive-like behaviour @PND56 ↑anxiety-like behaviour in the EPM was associated with ↑protein levels of TNFα and MCP-1; ↑CRHR2 and KMO mRNA expression; and ↓KAT1 mRNA expression in the hippocampus. No difference in immobility in the Forced Swim Test.
Wu et al. [42]	Rat, Sprague Dawley	HFD (26.2% protein, 34.9% fat, 26.3% carbohydrate) HPD (highly palatable diet) (16.8% protein, 4.8% fat, 74.3% carbohydrate) CD (20% protein, 7.0% fat, 64% carbohydrate)	Pre-mating Mating Gestation Lactation (12 weeks)	Male	Striatum (CP, dCP, vCP) NAc	Target behaviour: reversal learning @9–10 weeks old ↓ reversal learning and memory in visual discrimination and serial reversal learning test was associated with disrupted dopamine homeostasis (↓DAT in the striatum CP) and leptin signalling (↓pSTAT3 protein level in response to leptin injection) in both HFD and HPD offspring (no difference between HFD and HPD).
Zieba et al. [43]	Mouse, C57BL/6J	HFD (21% protein, 43% fat, 36% carbohydrate) CD (22% protein, 13% fat, 65% carbohydrate)	Mating Gestation Lactation (8 weeks)	Male	n/a	Target behaviour: Schizophrenia-like behaviour @38 weeks old No altered behavioural response in tests for cognition, sociability, locomotion and exploration (EPM, Social interaction, Y-maze, Novelty suppressed feeding, Prepulse inhibition, fear conditioning and OFT)
Moreton et al. [44]	Rat, Wistar	Cafeteria diet (average intake (g/d): 7.95 protein, 12.33 fat, 20.13 carbohydrates) + CD CD (average intake (g/d): 10.8 protein, 3.6 fat, 25.75 carbohydrate)	Lactation (3 weeks)	Male Female	PFC	Target behaviour: cognition @PND24–26 Offspring of cafeteria diet fed dams showed object recognition at Inter-trial interval (ITI) at 5 min but object recognition was impaired at ITI 30 min, compared to offspring of CD fed dams. ↓ DA, DOPAC, HVA and DOPAC/DA ratio in the PFC, in both sexes. ↑ 5-HIAA and 5-HIAA/5HT ratio in female PFC.
**Types of maternal overnutrition—high fructose/sucrose diet (Offspring was weaned on CD)**						
Erbas et al. [45]	Rat, Sprague Dawley	Fructose diet (30% fructose enriched water + regular chow) CD (tap water + regular chow)	Mating Gestation Lactation (18 weeks)	Male Female	Whole brain Hippocampus	Target behaviour: Autistic-like behaviour@PND50 Male offspring ↓sociability in social interaction test, ↑anxiety in OFT. ↑stereotypic behaviour and ↓ memory in passive avoidance test. The behavioural change in males was associated with ↑astroglial activity, ↓neuronal count in the hippocampal CA1 region, and protein concentrations of NGF, GAD67, NRG1 and 5HIAA ↓ in the whole brain. ↑TNFα protein concentration in the whole brain in both sexes.
He et al. [46]	Rat, Sprague Dawley	High sucrose (20% sucrose solution + standard chow) Control (tap water + standard chow) Standard chow contains 19.3% protein, 4.0% fat 39.1% carbohydrates.	Gestation (3 weeks)	Male	Hippocampus	Target behaviour: Learning and memory at 18 months old ↓spatial learning and memory in MWM was associated with ↓lipid peroxidation markers (TBARS and Catalase), NADPH2 and SOD1 in the hippocampus.
Wu et al. [47]	Rat, Sprague Dawley	High fructose diet contains 60% fructose) CD (unknown) Other macronutrient components unknown.	Gestation Lactation (6 weeks)	Female	Hippocampus	Target behaviour: spatial learning and memory @3 months ↓spatial learning and memory in MWM associated with ↑ nuclear HDAC4 and total HDAC4 expression; ↑DNA binding activity of HDAC4 to the BDNF promotor I, II, IV. ↓BDNF exon II and IV mRNA levels and ↓ total and free forms BDNF in the hippocampus,
Yamazaki et al. [48]	Rat, Sprague Dawley	Fructose diet (20% fructose water + CD) Glucose diet (20% glucose water + CD) CD (Tap water + CD)	Gestation Lactation (6 weeks)	Male	Hippocampus	Target behaviour: learning and memory @PND60 ↓hippocampal dependent cognition in NOR and fear conditioning, which was associated with ↓adult neurogenesis (BrdU/NeuN double positive cells); ↓BDNF ant TrkB mRNA expression, ↓ protein BDNF level, and ↑methylation of the BDNF promoter regions.
**Types of maternal overnutrition—high salt diet (Offspring was weaned on CD)**						
Digness et al. [49]	Rat, Wistar	High salt diet (29% protein, 14% fat, 58% carbohydrate, 4% Sodium chloride) Low salt diet (29% protein, 14% fat, 58% carbohydrate, 1% sodium chloride)	Gestation Lactation (6 weeks)	Male	Limbic PFC Infralimbic PFC NAc	Target behaviour: Stress sensitivity @PND21/60 No effect on reward-driven behaviours, including sucrose preference, conditioned place preference for cocaine, and forced swim stress-induced reinstatement of cocaine-induced conditioned place preference at PND 60, whereas changes in spine densities in the infralimbic PFC, the prelimbic PFC and the NAc shell at PND21 and 60

↑, increased; ↓, decreased; 5HIAA, 5-hydroxyindoleacetic acid; ACTH, adrenocorticotropic hormone; ARC, arcuate nucleus of the hypothalamus; CD, control diet; CP, Caudate-putamen; Crebbp, Creb binding protein; Crh, corticotropin releasing hormone; CRHR2, corticosteroid releasing hormone receptor 2; DA, dopamine; DAT, dopamine transporter; dCP, dorsal caudate putamen; Dnmt3, DNA methyltransferase 3; DOPAC, 3,4-Dihydroxyphenylacetic acid; EPM, elevated plus maze; FST, forced swim test; GAD67, glutamic acid decarboxylase 67; Gadd45b, DNA-damage-inducible 45 beta; GR, glucocorticoid receptor; HFD, high fat diet; HPA axis, hypothalamic-pituitary-adrenal axis; HVA, homovanillic acid; IkBa, I-kappa-B-alpha; IL1-Ra, interleukin-1 receptor antagonist; Insr, insulin receptor; KAT1, kynurenine aminotransferase; KMO, kynurenine mono oxygenase; Lepr, leptin receptor; MCP-1, monocyte chemoattractant protein 1; Mecp2, methyl CpG binding protein 2; MKP-1, mitogen-activated protein kinase phosphatase-1; MWM, Morris water maze; NAc, Nucleus Accumbens; NADH2, nicotinamide adenine dinucleotide phosphate oxidases 2; NfkB, nuclear factor kappa beta; NGF, nerve growth factor; NOR, novel object recognition; Nr3c1, glucocorticoid receptor; NRG1, neuregulin 1; OFT, open field test; PFC, prefrontal cortex; PND, postnatal day; PVN, paraventricular nucleus of the hypothalamus; SOD1, superoxide dismutase 1; TBARS, thiobarbituricacid reactive substance; TNFα, tumor necrosis factor alpha; USV, ultra sonic vocalisation; vCP, ventral caudate putamen; VMH, ventromedial hypothalamic nucleus of the hypothalamus; VTA, ventral tegmental area.

## Data Availability

Not applicable.
